# Tracking Resistance in Polymeric Insulation Materials for High-Voltage Electrical Mobility Applications Evaluated by Existing Test Methods: Identified Research Needs

**DOI:** 10.3390/polym15183717

**Published:** 2023-09-10

**Authors:** Jordi-Roger Riba, Manuel Moreno-Eguilaz, Santiago Bogarra

**Affiliations:** Electrical Engineering Department, Universitat Politècnica de Catalunya, 08222 Terrassa, Spain; manuel.moreno.eguilaz@upc.edu (M.M.-E.); santiago.bogarra@upc.edu (S.B.)

**Keywords:** insulation polymer materials, tracking resistance, electrical discharges, insulation degradation

## Abstract

With the increasing electrification of the transportation and mobility sectors, polymer insulation materials are inevitably exposed to harsher environments, including exposure to contamination, wide temperature ranges, operation at higher voltages and switching frequencies, and low-pressure environments. This paper reviews the tests to characterize the polymeric materials used in insulation systems for electric mobility applications, focusing on resistance to tracking. This paper also reports on the limitations of existing standard test methods and identifies the challenges and research needs to meet the increasing demands of the electric mobility industry. To this end, an evaluation of the scientific and technological state of the art is carried out through the analysis of theses, research articles, technical reports, manufacturers’ datasheets, international standards, and white papers.

## 1. Introduction

With the increasing electrification of the transportation and mobility sectors, polymer insulation materials are inevitably exposed to diverse environments, including wide temperature ranges, operation at higher voltages and switching frequencies, and low-pressure environments. Polymers are the most commonly used insulation materials due to their availability, reliability, competitive cost, and ease of manufacture. The selection of the most suitable polymer material depends on the operating conditions and requirements of the particular application. Technological advancements place increasing demands on insulation materials and systems for reliable operation under a wide range of environmental and operating conditions [1]. However, despite the leading role of insulation systems, a single point of insulation failure can be the cause of irreversible damage and catastrophic consequences to the electrical equipment in question [2].

The electrification of transportation is growing rapidly due to stringent international regulations on greenhouse gas emissions and because electric vehicles use energy more efficiently. Today’s battery electric vehicles operate at voltages in the range of 200–1000 V [3]. Aviation is also developing electrification plans, following the same trend as road vehicles. Aircraft electrification is believed to have several advantages, such as reducing weight, reducing equivalent CO_2_ emissions, increasing energy efficiency, improving reliability and safety, or facilitating maintenance operations [4]. It is estimated that future electric jetliners will require tens of MW of electrical power with maximum voltages ranging from 1000 V-DC [5] up to 20 kV, so the insulation system will need to withstand approximately 40 kV [6]. As aircraft move towards higher voltages, creepage, and clearance distances become more critical to limit the risk of electrical failure. However, conservative designs can lead to significant penalties in terms of power density [7], a key parameter in aircraft applications.

Due to the high compactness requirements typical of electric mobility systems, the insulation systems in electrical, electronic, and cabling systems must withstand high electrical stresses [8], which is very challenging from the point of view of the required insulation materials and poses many difficulties related to reliability requirements and minimizing the occurrence of insulation failures.

In the case of aircraft, the high compactness [9], high-altitude, low-pressure environment, condensation, or even increased levels of UV radiation or vibration make the design of insulation systems still more complex and challenging because they are not compatible with conventional terrestrial insulation systems [10]. In addition, the reduced air density characteristic of the aircraft environment significantly reduces the dielectric strength of the air [11,12], as well described in several references [13,14,15], and makes the thermal exchange more difficult [16]. 

Propulsion electric motors must be typically fed by electronic inverters switching at frequencies in the kHz range, even above 20 kHz [17]. Such propulsion motors will often operate in unpressurized environments, and low air densities are known to reduce partial discharge (PD) inception voltage [18]. The combined effect of high switching frequencies, low voltage rise d*v*/d*t,* and phenomena related to cable reflection exacerbate this problem [19], thus favoring the inception of surface and internal discharges in insulation systems and accelerating their degradation. Stator coils of high-voltage rotating machines typically contain copper conductors and insulation layers, which are often made of epoxy resin and planar mica flakes because of their excellent insulating and thermal properties, adjusted cost, and ease of use [20]. For rated voltages below 3 kV, enamel insulation is usually sufficient, but for inverter-fed machines rated at 3 kV and above, mica-based tapes are recommended due to the continuous repetitive pulses with high d*v*/d*t* values [21].

Polymeric insulators for outdoor high-voltage applications are often based on ceramics, glass, or polymeric materials such as silicone rubber filled with aluminum tri-hydroxide (ATH), which acts as an arc-quenching agent [22]. The addition of micro- and nano-sized ATH fillers to the silicone rubber matrix allows for improved resistance to erosion and tracking [23].

This new scenario is forcing the increased use of organic insulation materials in the mobility and transportation sectors, which are exposed to harsh environments. Therefore, the long-term behavior of polymeric insulating materials under different environmental and operating conditions should be investigated. Elements such as power electronic switching devices, circuit breakers, and lightning produce voltage surges in the surface of the organic insulation, leading to tracking failure [2], a type of dielectric breakdown that typically occurs on the surface of polymeric insulation due to the progressive formation of carbonized conductive paths due to the combined effects of electrolytic contamination and electrical stress [24]. These carbon paths are formed on the insulation surface by the decomposition of carbon products due to the cumulative effect of the discharges [25]. Tracking breakdown occurs when the carbon deposits or tracks form a continuous conductive path [26], so char formation plays a key role in this process [27]. In addition, repetitive power system operation is a major factor in tracking failure [28]. When the discharge is intense and prolonged, carbonization products are progressively formed on the polymer surface. When these products bridge the electrodes, there is an abrupt drop in insulation resistance [8]. A sudden drop in insulation resistance occurs when the carbonized deposits bridge the gap between the electrodes, thus leading to ignition, short circuit, and fire hazards [29,30]. Tracking failure is typical of organic insulators, so inorganic materials such as glass or ceramics do not undergo surface breakdown due to tracking issues.

Premature degradation of insulation systems is largely determined by partial discharge (PD) activity and poor thermal management [31]. PDs are discharges localized to a specific area of the insulation and do not completely bridge the separation between the electrodes and progressively age the insulation material [32]. Generating initial or seed-free electrons is crucial for the inception of PD activity in atmospheric air. When an intense electric field is present, it accelerates the free electron, eventually colliding with a neutral air molecule [33], thus initiating the discharge process. These collisions release more electrons that are accelerated by the electric field along the mean free path, the distance an electron travels on average before colliding with other particles. The mean free path in a gas is inversely proportional to the pressure, so the mean free path is larger at low pressure. The same is true for the average energy acquired by the electron as it travels along the mean free path. Therefore, in low-pressure gases, although there are fewer charge carriers due to the low-pressure environment, they acquire more energy and ionize more efficiently [34]. Therefore, for a given amplitude of the applied voltage, the PD activity increases at low-pressure operation, and the energy of the discharges increases as well as their potential damage [35,36]. As a result, insulation materials that may withstand moderate levels of PD activity at ground level may fail at cruising altitudes [37]. In addition, the sensitivity of conventional electromagnetic methods may be insufficient to detect PD activity at low pressures, as the amplitude of PD pulses typically decreases with pressure [35]. PD activity is typically detected using radio interference voltage (RIV) detectors, electromagnetic PD detectors, sound level meters [38] or radio frequency [39], and ultrahigh frequency antennas [40,41]. Surface PD activity can also be detected using optical methods [37,42,43,44], such as optical spectrophotometers [45], due to the ultraviolet (UV) and visible light emissions. 

Although corona-resistant wires can be used to provide additional protection against sporadic PDs, in [46], it was shown that at 15% of sea level pressure, corona-resistant wires could withstand the effect of electrical discharges for only a few seconds, so the aging mechanism could potentially cause the insulation to fail in short periods. According to [37], the behavior of corona-resistant wires based on polymers filled with inorganic microparticles or nanoparticles improves at ground level, but they could not ensure safe operation at reduced pressures, so extensive experimental designs and research work are still required to support such an assumption. 

The increase in voltage amplitude, combined with the decrease in conductor spacing and the increase in operating frequency due to the move to variable frequency generators in aircraft, increases the risk of arc ignition, which is potentially destructive and has resulted in several aircraft incidents and accidents [47]. The presence of foreign object debris (FOD) in insulation systems is also a factor to consider.

An early study [48] showed that polyimide (PI) insulation was susceptible to accelerated degradation when immersed in distilled water. A few years later, another study [49] concluded that the life of the wiring system in naval aircraft using Kapton PI insulation could not withstand the harsh conditions of naval aircraft, including the salty environment, chemical cleaners, severe changes in aircraft altitude, temperature, and humidity, or sustained wire vibration. Although Kapton PI insulation has excellent mechanical and dielectric properties, when exposed to water and chemical cleaners, the resulting chemical reaction tends to break the polymer chains, facilitating the formation of tracking and weakening the mechanical and dielectric strength [50], facilitating short circuits between adjacent electrical wires. Consequently, the electrical insulation system is a core technology for the electrified aircraft of the future [10].

The harsh environment of an aircraft presents many challenges to the design of the insulation system, a problem that is exacerbated by the wide range of pressures characteristic of aircraft, such that the insulation against PD decreases dramatically at low pressures.

Insulation-related failures are recognized as a potential cause of numerous aircraft incidents and accidents [10,51]. However, they still occur despite increased awareness of the problems associated with aircraft wiring systems, maintenance, and aging. With the increasing electrification of mobility systems, polymeric insulation materials are becoming even more important to ensure proper behavior and safe operation of the entire electrical system.

This paper reviews the effects that lead to tracking failures in polymeric materials used in insulation systems for electric mobility applications. It also reports on the limitations of existing standard test methods and identifies the challenges and research needs to meet the increasing demands of the electric mobility industry. To this end, an evaluation of the scientific and technological state of the art is performed by analyzing dissertations, research articles, technical reports, manufacturers’ datasheets, international standards, and white papers. The literature review presented here provides a critical and comprehensive analysis of the need for polymeric insulation materials and identifies the challenges and research needs.

The remainder of this paper is organized as follows. Section 2 describes the arc tracking phenomenon and its consequences. Section 3 develops the causes of polymer degradation, which can also facilitate arc tracking conditions. Section 4 describes the existing standard tests for evaluating the tracking resistance of polymer materials. Section 5 describes the role of the comparative tracking index on polymeric materials. Section 6 summarizes the identified research needs. Finally, Section 7 concludes this paper.

## 2. The Arc Tracking Phenomenon

PDs are localized discharges that do not completely bridge the insulation gap between the electrodes [52]. However, the impact of charge carriers, mainly electrons, at the PD sites causes chemical changes in the polymer material and damages its insulating capability. These chemical changes could create a conductive carbon path, allowing an electric current to flow with a consequent increase in temperature. These changes tend to cause premature insulation degradation, facilitating the formation of an arc and even complete insulation failure [53]. The mechanism for creating the arc via dry band formation is explained later. Because the molecular composition of most polymeric materials contains carbon atoms, they can be subject to tracking failure, the dielectric breakdown that can occur at their surface [54]. 

Electrical tracking refers to the electrical and thermal activity generated along conductive carbon paths in the surface of organic insulators due to surface PD activity, often triggered by the presence of moisture, liquids, dust, or debris, i.e., surface contamination [55,56,57]. Electrical tracking can also be initiated by mechanical means, such as insulation abrasion, chafing between different wires or between a wire and the structure of the vehicle, or even by short circuits [57]. 

Contaminated environments are known to facilitate the development of electrical discharges and the degradation of polymeric materials [58]. Significant leakage current can be generated on the contaminated wet surface of insulation materials, which can lead to Joule heating and the formation of dry band arcing (DBA) [59,60]. The PD activity at these contaminated sites generates an intense bombardment of electrons [61] with the consequent temperature rise, which contributes to breaking the polymer chains, degrading the insulation, and thus transitioning from insulating to conducting states due to energetic thermal shocks, ultimately producing carbonized conductive tracks. Therefore, the area of the carbon tracks tends to increase over time. Arc tracking is the propagation of the arc along nearby wires [62] due to thermal degradation of the insulation [63], which becomes conductive. This, in turn, raises the temperature of the insulation and causes further thermal degradation. Despite the small size of the arc, it can ignite the flammable gases that are produced on the surface of the insulation [64]. As the arcing activity continues, the new discharges expand the area of the existing conductive tracks, facilitating flashover or electrical breakdown [58], with the resulting fire hazard [65], with the resulting fire hazard [66]. The duration of the arcing phenomenon can range from a few tenths of a second to several seconds. It produces voltage and current in a wide spectral range [67]. Depending on the topology of the electrical network, arc tracking can result in a stabilized arc as long as the electrical protective devices are not tripped. The phenomenon may recur after the arc is extinguished and the system is reconnected [62].

Open harnesses, such as those found in the automotive and aircraft industries, are open and accessible. They have no overall protective covering, so if the insulation layer of one wire becomes thermally carbonized, it will affect the polymeric sheaths of the nearby wires, resulting in the complete failure of the bundle or even the entire harness [68]. This can cause a large current leakage, resulting in flashover [69] and severe loss of functionality [70]. Although arc tracking typically occurs in high-voltage systems, it has also been reported in low-voltage systems [57]. 

Arc behavior depends on the nature of the applied voltage. Whereas in AC circuits, the arc extinguishes at each zero crossing of the current, in DC circuits, the arc tends to self-extinguish unless a minimum current ensures arc stabilization. Under low-pressure conditions, the effects of the arc are more severe than under ambient pressure, but the arc is shorter in duration and extinguishes quickly after the first pass per zero current [71]. 

Therefore, in order to accurately size insulation systems for electrical equipment, it is essential to understand the mechanisms that lead to electrical tracking and the influence of environmental conditions [7].

## 3. Causes of Polymer Degradation

Polymeric materials are required in insulation systems for use in low, medium, and high-voltage applications for application in both standard and low-pressure environments. In the absence of oxidation, carbon deposits can form on the surface of the polymeric material, which can be decomposed by the action of dry-band arcing [28,72]. 

Polymer degradation is an undesirable process by which a polymer material loses its original properties when exposed to operational and environmental stresses [73]. There are different types of degradation, such as thermal, photochemical, mechanochemical, oxidative, polymer burning [74], and tracking [61]. Chemical degradation and electron bombardment can also lead to surface discharges and, ultimately, to tracking failure of polymeric materials [75].

Moisture and ionic contamination can cause ionic migration on the surface of polymeric materials, creating another potential failure mode and reducing the insulation resistance. This electrochemical and water-dependent phenomenon occurs under normal environmental conditions when the local current density and temperature are sufficiently low to allow the presence of an aqueous film on the surface of the polymer. Ionic migration can occur when the insulating material separating the conductors acquires sufficient moisture to allow ionic conduction in the presence of an electrical potential. The positive electrode, the metallic anode, is oxidized (Anode → Anode*^n^*^+^ + *n*e.) The electric field moves the positively charged ions through the moisture pathways toward the cathode (negative electrode). Water molecules dissociate at the cathode (H_2_O + e^−^ → 1/2H_2_ + OH^−^), allowing OH^−^ ions to drift toward the anode. The metal cations (Anode*^n^*^+^) can form oxide deposits that grow in filaments or dendrites [8]. 

Surface treatments can increase the resistance of polymers to arc tracking, and they have been successfully applied for this purpose [76], using additives to increase the hydrophobicity of silicone rubber.

### 3.1. The Effect of Air Density and Atmospheric Pressure

Air density greatly affects the effectiveness of surface discharges because atmospheric air is already present in the discharge process. Air density *ρ_air_* [kg·m^−3^] is directly related to the local air pressure *P* [Pa] and temperature *T* [K], as described in the ISO Standard Atmosphere [77], as,

(1) $$\rho_{air}=P/(R_{specific}T)$$

where *R_specific_* = *R/M* is the specific gas constant for dry air, where *R* = 8314.32 J/(kg·kmol) and *M* = 28.9644 kg/kmol is the molar mass of atmospheric air measured at sea level. 

According to Equation (1), air density is directly proportional to air pressure but inversely proportional to air temperature. 

The local electric field at the discharge site accelerates the electrons in the air released during the discharge process along the mean free path. The mean free path is defined as the average distance an electron travels before colliding with other particles [34] and can be approximately calculated as,

(2) $$\lambda =M/(\sigma N_{A}\rho_{air})$$

where *N_A_* = 6.022 × 10^23^ molecules/mol is the Avogadro’s number and *σ* = π(2*r*)^2^ [m^2^] is the collision cross-section of the air [78], where *r =* 2 × 10^−10^ m is the radius of the air molecules [79]. From Equation (2), it can be seen that the mean free path is inversely proportional to the air density. In addition, the mean kinetic energy *ε_m_* gained by an electron accelerated through the mean free path *λ* can be expressed as [34],

(3) $$\varepsilon_{m}=Eq_{0}M/(\sigma N_{A}\rho_{air})$$

where *q_0_* is the elementary charge, i.e., *q_0_* =1.602 × 10^−19^ C. As the air density decreases, the released electrons tend to accelerate faster, gain more kinetic energy along the mean free path, and ionize more efficiently. Therefore, at low air density, even at reduced discharge intensity with a small number of released electrons, they are more effective in producing ionization events than those produced by stronger discharge activity at normal pressure [35]. 

From Equations (2) and (3), it can be deduced that in unpressurized areas of aircraft systems, due to the lower air density, the mean free path and the kinetic energy acquired along the mean free path of the electrons released during the discharge increase, thus increasing the proportion of hot electrons, those with sufficient energy to ionize. From Equation (3), it can be deduced that the mean free path and the mean electron kinetic energy acquired along the mean free path increase linearly as 1/*ρ_air_*. Thus, for a given temperature, the mean free path at 20 kPa (the approximate pressure at which jetliners fly) is five times that corresponding at 100 kPa (sea level pressure).

The minimum voltage required to initiate surface discharges in the air increases with increasing ambient pressure, which also affects the tracking pattern [80]. Experimental studies on printed circuit boards indicate that low-pressure operating conditions delay tracking failure [61]. However, according to [54,81], the DC tracking resistance can increase, decrease, or remain almost unchanged as the atmospheric pressure decreases, so some insulation materials may exhibit inadequate behavior in high-altitude applications. It has also been found that the tracking resistance tends to be higher under AC supply than under DC supply [54].

Tracking experiments show two main types of behavior, i.e., erosion-type damage and tracking-type damage [82]. In these experiments, the uneven evaporation of water from the contaminant causes arcs to form over localized high-resistance areas of the insulation surface. These arcs generate high temperatures, and in materials susceptible to tracking, carbonization can occur in the direction of the electric field, ultimately leading to insulation failure. However, many insulation materials, such as PTFE, do not form carbon tracks. Instead, the arc gradually causes localized erosion (the loss of material by electrical discharge or leakage current [55]) by depolymerization, which produces a non-flammable gas. Polymeric materials that do not form carbon tracks will have their surface progressively eroded by the gaseous discharge, eventually leading to dielectric breakdown due to the erosive effect of the discharge [54,82]. In other insulation systems containing mica flakes or ATH, polymer decomposition is prevented from forming a continuous carbonaceous path.

The CTI of polymeric insulation materials depends on atmospheric pressure. However, this dependence depends on the type of material, i.e., while for some materials, the CTI increases with atmospheric pressure, others show an inverse behavior, while for others, the CTI is almost independent of atmospheric pressure [54]. Therefore, polymeric materials can be classified into three types of behavior when analyzing CTI or tracking resistance as a function of pressure. In polymer materials such as polycarbonate and paper-based phenolic laminates, the tracking resistance increases with decreasing ambient pressure. In materials such as polybutylene terephthalate, the resistance decreases with ambient pressure. Finally, in materials such as epoxy resin, the tracking resistance is almost independent of pressure [54]. It should be noted that this different tracking behavior with the atmospheric pressure of different polymer materials is not related to the reduction in the dielectric strength of air at lower values of atmospheric pressure.

It has been reported that organic insulation materials with an oxygen index (the minimum concentration of O_2_, expressed as a volume percent, that supports flaming combustion of the polymer measured by passing a mixture of O_2_ and N_2_ over a burning sample) of less than 21 tend to undergo tracking degradation when the atmospheric pressure is reduced because, at reduced atmospheric pressure, the supply of oxygen is insufficient [9]. As a result, the free carbons tend to remain on the surface of the insulation material, and as the pressure decreases, the depth of erosion and weight loss increases. In addition, materials with a low heat deflection temperature have a low resistance to tracking, so it is easier to undergo tracking degradation at higher temperatures. In the case of polyethylene, the tracking resistance under applied AC voltage is lower for both weight loss and erosion depth, indicating potential reliability and safety risks when AC tracking resistance results are used to design an insulation system for DC voltage application. In [9], it is also reported that the ground plane data for DC tracking resistance cannot be used for high-altitude or high-temperature applications.

At low pressure, an insufficient supply of oxygen can reduce the rate of oxidation on the polymer surface [61]. However, as discussed earlier, the dielectric strength of air decreases at low pressure. In addition, the convective heat transfer coefficient decreases, so convective cooling is less effective at low pressure [83] because there is less air. The balance between reduced oxygen supply, lower dielectric strength of air, and reduced effectiveness of convective cooling determines the rate of carbonization of the polymer surface. Tracking failure is greatly influenced by the thermal energy involved in the discharge, which in turn depends on the oxidation rate, surface temperature, and surface discharge rate [61]. Surface temperature plays a key role in tracking failure by accelerating the thermal decomposition of the carbonaceous char in the track path [61]. 

Therefore, to improve the reliability and safety of electrical insulation, it is essential to develop research plans on resistance to tracking. In particular, for low-pressure applications, it must be confirmed whether the resistance to tracking of polymeric materials is different from that at ground level [54].

### 3.2. The Effect of Temperature

One of the major concerns with polymeric insulation materials is their life expectancy, especially in outdoor environments, which results in thermal [84], mechanical and dielectric degradation [85] over time. In a study conducted on polymer films [86], it was shown that for a given film thickness, the aging rate increases with the ambient temperature due to the higher mobility of the charge carriers. 

Dry band arcing (DBA) generated in polymeric materials induces high surface temperatures that can lead to thermal degradation. Although corona discharges also occur during standardized tests to evaluate the tracking resistance of insulation materials, their influence on thermal degradation is small [87]. Thermal images showed that the discharges caused a rapid temperature rise on the sample, with the temperature at the center of the carbon track exceeding 1200 °C, while the temperature on most of the sample surface was above 150 °C, causing the electrolyte solution to evaporate. These extreme conditions and the rapid temperature rise convert carbon atoms into residual char and create a shortage of oxygen, which in turn promotes tracking conditions [87]. 

In [88], the thermal degradation and temperature rise at the surface of composite insulation due to low-frequency corona discharges were studied, and it was concluded that corona discharges can cause microscopic degradation, although no visual degradation was observed.

Tests performed according to the IEC 60587 [55] using the inclined plane test show that during the arcing process, the temperature in the central path of the track can exceed 1200 °C, while the temperature on the surface of the sample can exceed 150 °C so that the electrolyte supplied by the top electrode tends to evaporate. The polymer sample subjected to thermal stress generates various gases, some of which are rich in carbon. The high energy released can initiate thermal depolymerization (the process that converts a polymer into component monomers) of the polymeric material, forming carbon tracks by breaking the C-H chemical bonds on the interelectrode surface [89]. At very high surface temperatures, carbon atoms in such gases and atmospheric CO_2_ can be converted into conductive carbon [90]. The conductive carbon regions enhance the electric field around the carbon track and, in contaminated and electrically stressed environments, generate more wetting-induced DBA activity and trigger thermal decomposition and erosion of the untracked region. When wetted, the contaminant layer tends to form a conductive layer, allowing current to flow and eventually induce more DBA. Prolonged DBA activity causes Joule heating, generating an abrupt temperature rise that erodes the polymer material [90]. This cycle can be repeated, creating a discharge active region [87]. Such effects are of great concern because they limit the long-term life of polymeric materials [90].

In [91], it was shown that the sample temperature significantly affects the DC tracking of organic insulating materials, although the response to temperature depends on each specific material. It was also shown that the tracking degradation is caused by the carbon decomposed on the surface of the sample due to the temperature rise during the discharge process, which produces localized DBA on the surface of a sample due to the evaporation of the electrolyte solution and the posterior degradation produced by the scintillation discharge across the dry band.

Infrared thermal analysis studies have concluded that the tracking resistance of polymer samples decreases significantly once the surface temperature exceeds a critical value. Therefore, materials with improved thermal conductivity and thermal stability show improved carbonized tracking life and better protection against surface discharges [89].

### 3.3. The Effect of Liquid Contaminants

Liquid contaminants play a critical role in the formation of carbon deposits. When the temperature of the liquid contaminant reaches the boiling point, it evaporates at a higher rate, facilitating the tracking process. Therefore, the ambient temperature and pressure (both play a key role in reaching the boiling point) and the electrical conductivity of the contaminant must be considered in tracking studies [7]. For example, the boiling point of water is 99.7 °C at 100 kPa, but less than 50 °C at 10 kPa. The conductivity of the liquid contaminant also changes with temperature because any increase in temperature decreases its viscosity and increases the mobility of the ions in the solution. An increase in temperature can also cause an increase in the number of ions in the solution. As a result, the electrical conductivity of the solution increases with its temperature (and time). Thus, pressure, surface temperature, and time affect the behavior of the tracking phenomenon.

The presence of a liquid contaminant on the surface of an insulating material subjected to an intense electric field induces an electric current to flow through the contaminant, causing it to heat up. As a result, some of the contaminant evaporates first, forming a dry band of high electrical resistance. Due to the high resistance, the current across this region will produce a significant voltage drop, and as the dry band grows, the likelihood of an electrical event occurring in this region increases. The amplitude and nature of the applied voltage (AC, DC, pulsed, etc.), the environmental conditions (temperature and pressure), and the rate of evaporation will largely determine the duration of the arcing event. If the surface is repeatedly exposed to moisture, the process may start again. These repeated effects can cause significant insulation damage and carbonization, resulting in eventual dielectric failure [7].

In [7], it is reported that for glass fiber-reinforced epoxy resin (FR4), which is widely used for printed circuit boards (PCBs), the voltage required to initiate tracking decreases with ambient pressure, and the damage seen is more severe at lower pressures. 

### 3.4. The Effect of Dissociative Electron Attachment (DEA) 

DEA plays a key role in low-energy plasmas, i.e., ionized gases with an overall neutral charge [92]. Gas molecules dissociate due to the attachment of incoming low-energy electrons (AB + e^−^ → A + B^−^) [93]. Because DEA disrupts polymeric bonds and causes localized insulation degradation, DEA degradation is a major determinant of the damage caused by electrical discharges in polymeric materials [37]. The DEA activity in various polymeric materials tends to dissociate the C-H bonds of polymer chains, forming free radicals and H^−^ ions that locally degrade the polymer lattice. At least 8 eV are required to dissociate C-H bonds [94] (4 eV according to [37,95]), so only the hot electrons (high-energy electrons) with an energy above this limit value can damage the surface of the polymer insulation [96]. The energy of the hot electrons is much lower than that required to initiate breakdown activity in the solid dielectric. This is because the dielectric constant of the polymer is higher than that of the surrounding air, so the electric field strength in the solid dielectric is lower. Therefore, the hot electrons undergo rapid thermalization by DEA of C–H bonds and impact ionization. These cumulative effects can eventually cause irreversible insulation degradation [97].

## 4. Tests to Evaluate the Tracking Resistance of Polymer Materials

There are several standardized tests to determine the resistance of polymeric insulation for wires to dry and wet tracking, the most commonly used of which are summarized in the following subsections.

### 4.1. Dry Arc Tracking Tests

Dry arc tracking tests are not particularly useful for assessing the performance of insulation materials for outdoor applications or exposure to moisture because they do not use any type of contamination [28].

#### 4.1.1. Dry Arc Tracking Test according to the ASTM D495

**Figure 1:**
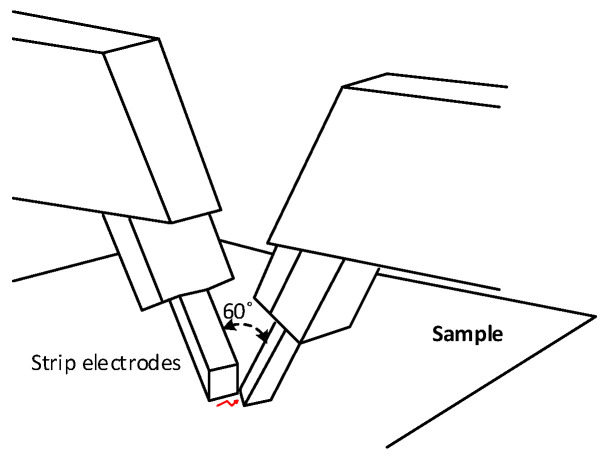
Strip electrodes used in the ASTM D495 dry arc test.

The ASTM D495 [98] describes the test methods for determining the dry arc resistance of solid electrical insulation. The tests are performed by applying 12,500 V-AC at the power frequency between the electrodes shown in Figure 1 and a current of 10 mA. The strip electrodes, which can be made of tungsten or stainless steel, must be positioned in the same vertical plane and inclined 35° from the horizontal. 

The test ends when conductive paths are formed over the entire surface of the specimen, the arc extinguishes, and there is a noticeable change in sound. The results of this test method are severely limited by many limitations, such as the dependence of the results on the bar material, changes in the current, changes in the timing of the intermittent arc, or other changes that affect the nature of the discharge.

#### 4.1.2. Dry Arc Tracking Test according to the UL 746A 

**Figure 2:**
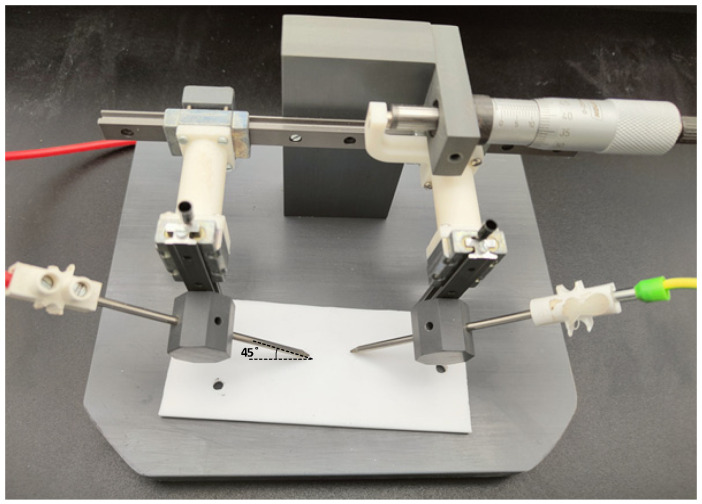
Steel rod electrodes used in the UL 746A dry arc tests.

The UL 746A standard [99] has a section on dry tracking high voltage arc resistance to ignition for polymeric materials. This test is designed to determine the ability of the surface of the insulating material to form a visible carbonized path or resist ignition when subjected to a low-current, high-voltage arc. UL 746A suggests using 303 stainless steel rod electrodes with an outer diameter of 3.2 mm and a length of approximately 102 mm. The tip is machined to a symmetrical conical point at an angle of 30°. Figure 2 shows the layout suggested by the UL 746A standard.

The electrodes must be spaced 4 mm from tip to tip, and the circuit must be energized at the line frequency AC voltage. 

### 4.2. Wet Arc Tracking Tests 

This section summarizes different wet arc tests found in international standards.

#### 4.2.1. Wet Arc Tracking Test with Notched Wires according to the EN 3475-603:2018 Standard

**Figure 3:**
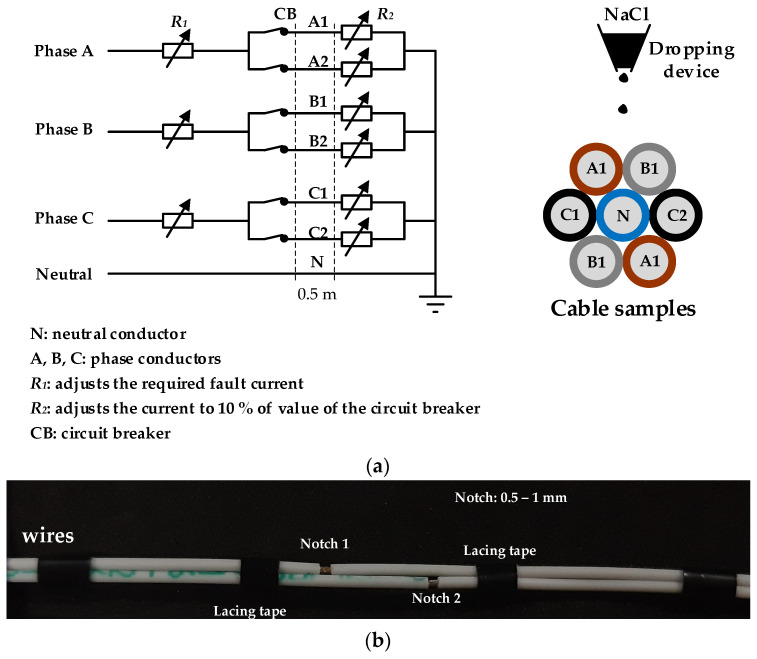
(**a**) Wet arc test for aerospace cables using seven straight and parallel cables inclined 10° from the horizontal plane, each approximately 0.5 m long. (**b**) Detail of artificially damaged wires. The cut penetrates the conductor around the full circumference of the wire and is 0.5–1.0 mm wide.

The standard EN 3475-603:2018 [100] evaluates the resistance to wet arc tracking applied to aerospace cables used in aircraft and must be used in conjunction with the standard EN 3475-100:2010 [101]. EN 3475-603:2018 describes the methods for assessing the behavior of wire insulation under the effect of an electric arc created and maintained between live wires maintained and initiated by a contaminating fluid. It applies to 115 V-AC systems operating at 400 Hz, although it also proposes conditions for 230 V-AC systems. It requires an electrolyte delivery system (3 ± 0.5% by weight of NaCl in distilled water) that delivers a constant rate of 100 ± 10 mg/minute, selecting a needle that delivers this flow rate in 6–10 drops/minute. The test will run continuously for 8 h or until the circuit breaker automatically trips. The parallel wires to be tested shall be artificially damaged by stripping a portion of the insulation, as shown in Figure 3. The dropping device must ensure that the drops hit the stripped portion of the insulation. The conductive cores of the various wires shall be connected to the phases and neutral of the electrical system. The specimen must be supported in free air and inclined at an angle of 10° to the horizontal. The electrical input connections must be at the higher end of the wires. The test is repeated three times for each cable size.

#### 4.2.2. Horizontal Plane Test according to the IEC 60112 Standard

**Figure 4:**
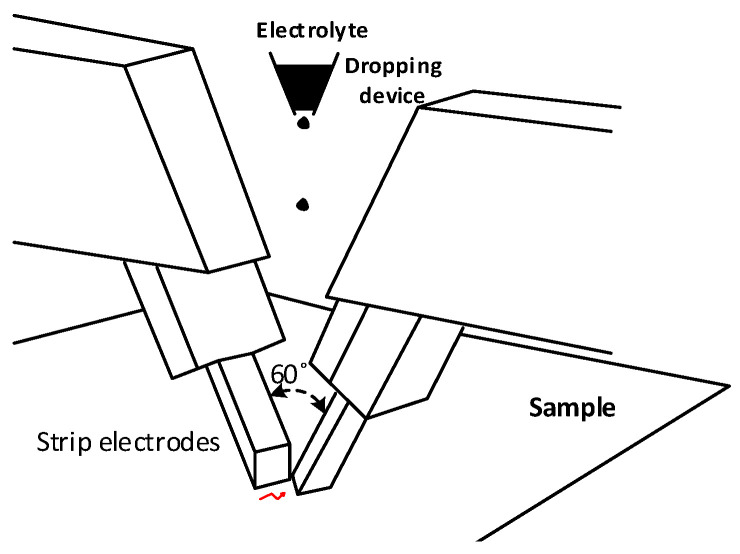
Wet arc test using strip electrodes.

IEC 60112 [24] proposes a horizontal plane test to determine the CTI of solid insulators. It is noted that the definition of CTI and its importance are found in Section 5. An electrolytic solution is used to simulate environmental contamination. A series of drops of electrolyte (0.1% NH_4_Cl and 0.5% by mass C_18_H_23_NaO_3_S in de-ionized water to give a resistivity of 3.95 ± 0.05 Ωm at 23 °C) is applied to the surface between the electrodes (see Figure 4) until either the overcurrent device operates, or until a sustained flame occurs, or until the test time has elapsed. During the test, an AC power frequency voltage in the range of 100–600 V-AC is applied between two platinum electrodes of a rectangular cross-section with the end in contact with the sample chisel edged at an angle of 30°. A dropping device is installed perpendicular to the axis of the electrodes. A total of 50 or 100 drops of the test solution must fall on the surface of the sample at intervals of 30 ± 5 s. The total mass of the 50 drops must be in the range of 0.997–1.147 g.

#### 4.2.3. Inclined Plane Test according to the IEC 60587 and ASTM D2303-13 Standards

The inclined plane test is well described in the international standards IEC 60587 [55] and ASTM D2303-13 [102]. They are defined as wet tests because they evaluate the tracking performance of solid samples under the action of a liquid contaminant (0.1 % by mass of NH_4_Cl and 0.02% by mass of a nonionic wetting agent in distilled or de-ionized water to obtain a resistivity of 3.85 ± 0.15 Ωm at 23 °C according to the ASTM D2303-13, or 3.95 ± 0.05 Ωm at 23 °C according to the IEC 60587). These tests are performed under AC power frequency supply and at atmospheric pressure and allow determination of both tracking and erosion resistance. Due to the inherent variability of these standardized tests, five specimens of each material must be tested for each determination.

**Figure 5:**
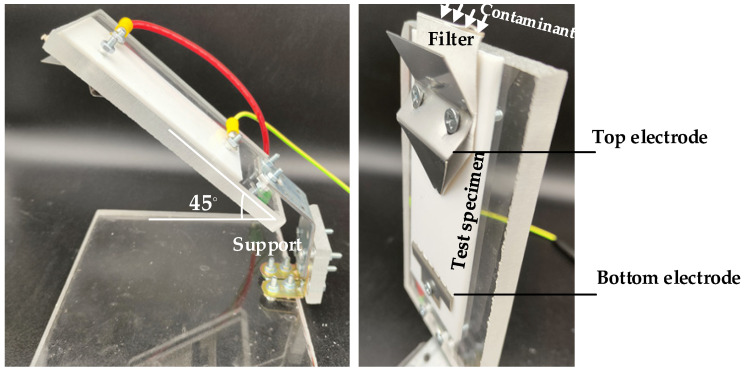
Inclined plane test.

As shown in Figure 5, the specimens are inclined at 45° from the horizontal while attached to electrodes at the top and bottom. The specified voltage is applied between the electrodes as the electrolyte flows from the top electrode to the bottom electrode. Tracking begins at the bottom electrode and progresses upward.

The contaminant must flow uniformly at the specified rate according to the values specified in the standards to ensure effective scintillation. The IEC 60587 and ASTM D2303-13 standards propose two types of tests.

In the step voltage test, the applied voltage is increased in 250 V steps. Each voltage level is held for 1 h, and if no failure is observed, the voltage is increased again by 250 V. This process is repeated until failure is observed, i.e., progressive tracking begins. 

In the constant voltage test, the applied voltage is held constant throughout the test, and the tracking time is recorded. Materials are classified according to the time required to develop tracking, although the time specifications differ between IEC 60587 and ASTM D2303-13. 

It is known that the dry band discharges (DBDs) generated in the inclined plane test precede different stages [103]. Initially, DBDs in the form of streamer discharges and partial arcs tend to remain in the same position until complete evaporation of the contaminant. Next, the surface resistance of the material decreases due to the carbonization process, causing DBDs to occur more rapidly and change their location. Finally, the leakage current increases because the discharges are longer and emit more light.

**Figure 6:**
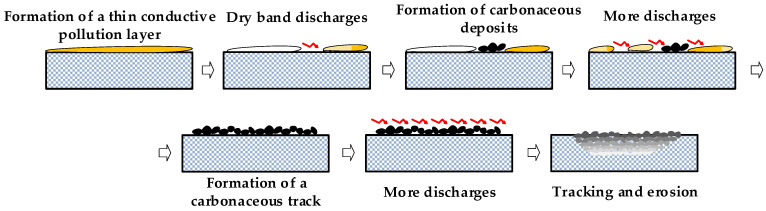
Schematic of the tracking and erosion process.

Figure 6 shows a simplified view of the tracking and erosion process.

## 5. The Importance of CTI for Polymeric Materials

According to the IEC 60050-212 [104] and the IEC 60112 [24], the CTI is defined as the numerical value of the maximum voltage, expressed in volts, that five specimens of material can withstand under specified test conditions without showing tracking failure and without showing flame persistence after 50 drops. Surface contamination, especially electrolytic surface contamination, is known to have a negative effect on CTI. Therefore, the CTI value is an indication of an insulator’s resistance to environmental influences when exposed to voltage. CTI is correlated with the tendency of materials to carbonize [28]. However, there are few papers on CTI in the technical literature [105]. Some of them study the influence of pressure [54,81], temperature [91], and atmospheric conditions [106] for some polymeric materials. 

Electrical insulation materials require a high comparative tracking index (CTI) [27]. The CTI value is used by certification and testing laboratories to assess the electrical safety of electrical equipment. According to IEC 60112 [24], the CTI is the most commonly used index to estimate the material’s sensitivity to surface tracking. The CTI value is determined from an accelerated test method [105], often conducted under contaminated conditions, where a voltage is applied between two electrodes placed on the surface of the material. The CTI indicates the tracking performance of insulation materials under wet and contaminated conditions. The total energy involved in the discharges is a critical factor in determining the tracking characteristics of the polymer material [54], but tests to determine the CTI typically have a high standard deviation because they are affected by the apparatus and environmental conditions [27].

The sensitivity to surface tracking can be measured by means of other approaches. For example, another systematic method based on the leakage current measurement has been proposed in [107]. This approach proposes to consider the critical AC voltage as the minimum voltage value that distorts the leakage current waveform or saturates the leakage current magnitude since these conditions indicate equilibrium between liquid contaminant replacement and evaporation [108]. 

Both the ASTM D3638 [109] and IEC 60112 [24] standards evaluate the comparative tracking index (CTI) over a short period of time under an AC low-voltage power supply in the presence of aqueous contaminants (NH_4_Cl solution). For this purpose, the surface of the test specimen is exposed every 30 s to a low voltage AC combined with a low amplitude electric current (typically less than 1 A) induced by the dropping of an aqueous contaminant. The applied voltage is in the range of 100–600 V-AC because below 100 V-AC, the NH_4_Cl solution does not evaporate between successive drops, and it is limited to 600 V-AC because higher voltages could cause air breakdown between the electrodes instead of along the surface of the sample under test.

This test requires the use of platinum strip electrodes, as shown in Figure 4. The voltage across these electrodes is maintained until the current flowing between them exceeds a predetermined failure value. The electrical resistivity of the aqueous NH_4_Cl solution must be 3.85 ± 0.05 Ωm at 23 °C according to the ASTM D3638 or 3.95 ± 0.05 Ωm at 23 °C according to the IEC 60112 standard. 

Most standards related to tracking resistance for polymer insulation materials deal with AC supply, so there is a lack of studies related to DC tracking resistance [54,110]. In [54], it was shown that the change in the number of drops required for tracking failure is directly related to the ambient pressure. Therefore, more research is needed to ensure the safe use of polymeric insulation materials in low-pressure environments, as it needs to be confirmed whether the tracking resistance of polymeric materials in low-pressure environments differs from that at standard pressure [54].

According to the IEC 60601-1:2005 standard [111], which applies to medical products, material groups are classified according to their CTI value as Group I (600 ≤ CTI), Group II (400 ≤ CTI < 600), Group IIIa (175 ≤ CTI < 400) and Group IIIb (100 ≤ CTI < 175).

The UL 746A standard [99] classifies the materials into six categories according to the comparative tracking performance level category (PLC), which is directly related to the CTI. While category PLC = 0 corresponds to CTI ≥ 600, category PLC = 5 corresponds to 0 ≤ CTI ≤ 100. 

Table 1 shows indicative CTI values for various polymer materials used in electric mobility applications obtained from different sources.

### 5.1. The Importance of the CTI Value

New applications in the field of electromobility require a high degree of compactness as they need to handle increasing electrical power in smaller volumes. Due to weight requirements, achieving higher power in smaller volumes requires higher operating voltages, so DC buses of 800 V and even 1000 V are now common [3,122] with switching frequencies above 30 kHz [123]. As a result, today’s insulation systems are subjected to greater electrical stress than classical applications based on 230/400 V-AC at a frequency of 50 Hz. Since air is a poorer insulator than polymer materials, surface discharges can occur on the outer surface of the polymer in contact with atmospheric air. This effect is amplified during low-pressure operation because the dielectric strength of air decreases at lower pressures and during high-frequency operation because the dielectric strength of air at approximately 1 MHz is 80% of that at 50 Hz [124]. Therefore, the interface at the boundary between the atmospheric air and the insulation is a weak point due to the lower dielectric strength of air compared to that of the insulation material. 

The tracking resistance of an insulation material is an indicator of how resistant its surface is to environmental factors from an insulation standpoint. The comparative tracking index (CTI) of an insulation material is a measure of the tendency of the material to form conductive tracks on its surface. However, an absolute measure of erosion and tracking resistance is not possible [54] because standard erosion and tracking tests only provide a relative ranking of insulation materials. 

The CTI test (also known as the wet tracking test) is described in the IEC 60112 standard [24] and is considered superior [108] to the dry arc resistance test described in the ASTM D495 standard [98].

The higher the voltage that must be applied during the test to form the conductive tracks, the higher the CTI value of the material. Therefore, electrical insulators require a high CTI value [27]. The insulating capability of the material tends to degrade slowly due to various environmental factors and surface conditions. The surface of the insulation can be contaminated during assembly and can be dusted and abraded during normal operation. These factors, combined with moisture (condensation) or liquid contamination, can significantly reduce the surface resistance of insulation materials. These conditions, together with the application of a voltage of sufficient amplitude in the presence of condensation or technical fluids, favor the formation of conductive paths along the particles deposited on the surface of the material. The induced electric current will gradually carbonize the path on the surface of the material through which the current flows.

### 5.2. Use of the CTI Value in the Design Phase of Electrical Devices

With the increasing voltage levels in electric mobility applications and the increasing use of high frequencies, the surface condition of insulation materials is becoming increasingly important as the formation of tracking paths inevitably leads to premature failure of components, equipment, and entire systems. The CTI allows the identification of a suitable insulation material based on standard specifications and intended use. The CTI value provides an overview of the materials that can be used in a design and allows the most appropriate material to be selected. CTI is also a measure of a material’s tendency to track under conditions of high stress and contamination.

The CTI value largely determines the minimum creepage distance (the shortest distance between two conductive paths measured along the surface of solid insulation) across the surface of an insulating material between a high voltage and ground electrode, so that a higher CTI value means a lower creepage distance, allowing the safe distance between two conductive parts to be reduced. Maintaining clearances in accordance with CTI reduces the risk of fire. There is a positive relationship between CTI and insulation performance, i.e., the higher the CTI, the better the insulation. For example, a material with a Group IIIa CTI will require a longer creepage distance than a material with a Group I CTI, regardless of the level of contamination. Therefore, the CTI has a significant impact on space requirements, so lower CTI values reduce the required creepage distance, which is of particular interest in electric mobility applications where space is limited. For this reason, the CTI value of insulation materials is of great importance in designing electric vehicles based on high-voltage technology.

## 6. Identified Research Needs

Polymeric insulation materials for electric mobility applications face several challenges related to the specific requirements of this application, such as high compactness and increased electrical stresses, wide temperature range, different voltage types (DC, AC, or switching mode operation), presence of dirt, condensation, and technical fluids, or low-pressure operation in the case of aircraft systems. Tracking resistance is particularly important in electric mobility applications due to the extreme environmental conditions and electrical stresses they must withstand. A new generation of insulation materials is needed to meet the pressing demands for downsizing and performance improvement [125]. 

Due to the important role of insulation systems in electric mobility applications and the rapid evolution and demanding requirements, we have identified several areas where research and development are needed:

- The CTI index has become the industry standard for measuring the dielectric strength of polymer materials. However, current standards for measuring CTI are limited to a maximum voltage of 600 V-AC, so they can only measure the tracking resistance of insulation materials at relatively low voltages. In addition, standardized tracking tests are based on line frequency (50/60 Hz) or 400 Hz for aircraft systems. The new requirements of electric mobility applications, which often involve switching power electronics and higher voltage levels, necessitate that tracking standards be adapted to the increased voltage levels and frequency range.
- There is a need to develop heat-resistant materials with extremely high tracking resistivity and proven effectiveness at voltages above 600 V (the current limit of the CTI standards) because they can reduce creepage between electrical conductors while allowing the manufacture of compacter and lighter electrical equipment for high-voltage applications. It seems that the tracking and erosion performance of aromatic polymers can be improved by means of organic-inorganic hybrid materials, as suggested in various references [126,127,128]. In [126], it was shown that PI/SO_2_ hybrid foams exhibit improved atomic oxygen (AO) erosion resistance, while [128] analyzed PI/SiO_2_ hybrid nanocomposites achieving similar results and concluding that such materials are promising candidates for aerospace applications, among others. Therefore, more research is needed in this area.
- Bus voltages in the electric mobility sector are increasing rapidly, so voltages of 800 V-DC and 1000 V-DC are common today, although even higher voltages up to several kV are expected in the future, especially for aerospace applications. As a result, there is a lack of tests to determine the DC resistance during tracking [9] or during switching operation. In the absence of international standards for evaluating erosion and tracking for DC applications, researchers often adapt or modify the existing standard AC tests for DC voltage conditions, although some inconsistencies have been reported [129]. Further research is needed in this area to adapt current standards to new high-voltage DC requirements or to develop new standards.
- It has been reported that the damage caused by positive and negative polarity DC tests is different [130]. Therefore, more research is needed in this area, and the standards will have to be adapted to take this aspect into account.
- It appears that the potential ionization efficiency tends to increase with reduced air density and pressure operation [35], and so does the potential damage to the insulation, at least for some polymer materials. Therefore, surface insulation failure data at standard atmospheric pressure may be inadequate for low-pressure applications [61]. There is a need for more data and experimental plans for low-pressure applications, which will be helpful in the development of future guidelines and standards.
- It is important to know whether tracking activity and severity are different at reduced air density (high altitude and low-pressure environments typical of aircraft systems) compared to standard air density [61]. Experimental data presented in [35], analyzing the discharge temperature, electron density, and electrical energy involved in the discharge process, suggest that both pressure and supply frequency have an important effect, such that a combination of high frequency and low pressure is the worst case. It is important to develop comprehensive test plans to determine the effects of existing insulation materials. The results obtained could be of particular interest for the selection of the most suitable materials for each application and could serve as guidelines for research into innovative materials.
- There is a need to develop standard tracking resistance tests that simulate the environmental conditions found in aircraft systems. In particular, the lower air density in unpressurized areas plays a key role. However, today’s standard tests do not account for this critical parameter.
- Due to the need for thinner insulation due to overall mass constraints, electric mobility systems require thin insulation layers with higher dielectric strength [131] that can withstand harsh environmental conditions (temperature, pressure, and humidity) and the presence of aqueous contaminants, so significant research efforts are needed in this area.
- Due to the limited number of studies that consider the effects of different parameters, such as temperature, UV exposure, or atmospheric pressure, more research is needed in this area.
- The results of the CTI test, although meaningful, are typically affected by a high standard deviation that is highly dependent on the equipment used and the environmental conditions [27]. Therefore, there is much concern about its repeatability (even for samples from the same sheet of material tested in the same laboratory) and accuracy [132], so more research plans are needed in this area.
- International standards typically use platinum, stainless steel, or copper electrodes. While platinum is very chemically stable, copper is more chemically active. However, other electrode materials may be used in real applications, so it is important to know the expected CTI value with the specific electrode material to be used in the final application [132]. There is a need for more experimental data to develop guidelines on how to address this issue.
- Studies of the combined effects of air density, temperature, and frequency on the tracking resistance and aging of polymeric materials are also needed.

## 7. Conclusions

The electric mobility sector is undergoing rapid changes in terms of requirements for weight reduction, high compactness, high voltage operation, fuel consumption, and greenhouse gas emissions. These demanding requirements lead to high electrical stresses on the insulation materials, thus requiring new developments and research in this area. 

This paper has reviewed the standard tests used to characterize the polymeric materials used in insulation systems for electric mobility applications, with a focus on tracking resistance. To this end, recent developments in the electric mobility sector have led to new requirements in terms of insulation material performance and endurance to tracking under high electrical stress conditions. Therefore, the paper has also reported on the limitations of existing standard test methods and identified the challenges and research needs to meet the increasing demands of the electric mobility industry. Various research needs have been identified, such as the need to thoroughly analyze the combined effects of air density, temperature, and frequency on the tracking resistance and aging of polymeric materials or to develop standard tracking resistance tests that simulate the environmental conditions found in aircraft systems. The higher voltage levels required by current and future electric mobility systems, which include DC applications with positive and negative DC buses, or the higher frequency range involved in the operation of switching power electronics, open up new areas of research as there is a need to adapt current international standards for tracking and insulation resistance. Other research needs include the development of thinner insulations, studies to better understand the role of the electrode material, or the development of tracking tests with higher repeatability than the current ones. These research needs are challenging because they require interdisciplinary teams involving different scientific disciplines, such as materials science, chemistry, or electrical, electronic, and aerospace engineering. 

## Figures and Tables

**Table 1:** Indicative CTI values of various polymeric materials used in automotive and aircraft applications.

Material	CTI value	Reference
Polyethylene (PE)	>600	[112]
Polytetrafluoroethylene (PTFE)	>600	[112]
Polymethyl methacrylate (PMMA)	>600	[113]
Polypropylene (PP)	>600	[114]
Polyamide (PA)	>600	[114]
Perfluoroalkoxy (PFA)	>600	[114]
Fluorinated ethylene-propylene (FEP)	>600	[114]
Silicone rubber (SiR)	>600	[115]
Polyvinyl chloride (PVC)	600	[116]
Acrylonitrile butadiene styrene (ABS)	600	[117]
Polyester resin	600	[112]
Ethylene tetraflurorethylene (ETFE)	575 ≤ CTI < 600	[118]
Polybutylene terephthalate (PBT)	500	[112]
Ethylene propylene diene monomer (EPDM)	415	[119]
Polyethylene naphthalate (PEN)	400 ≤ CTI < 600	[114]
Polystyrene (PS)	400 ≤ CTI < 600	[120]
Non-brominated epoxy resin	400 ≤ CTI < 600	[121]
Brominated epoxy resin	CTI ≥ 200 to > 600	[121]
Polycarbonate (PC)	175 ≤ CTI < 400	[120]
Polyphenylene sulfide (PPS)	175 ≤ CTI < 250	[114]
Glass fiber reinforced epoxy resin, PCB base material (FR4)	175 ≤ CTI < 250	[112]
Polyether ether ketone (PEEK)	175	[114]
Polyimide (PI), Kapton^®^	150	[112]
Phenolic resin (PF)	125	[112]

## Data Availability

Not applicable.
